# The Satisfaction Rate among Patients and Surgeons after Periareolar Surgical Approach to Gynecomastia along with Liposuction

**Published:** 2016-09

**Authors:** Ahmad Reza Taheri, Mohamad Reza Farahvash, Hamid Reza Fathi, Koorosh Ghanbarzadeh, Bijan Faridniya

**Affiliations:** Department of Plastic Surgery, Tehran University of Medical Sciences, Tehran, Iran

**Keywords:** Gynecomastia, Periareolar incision, Liposuction, Satisfaction rate

## Abstract

**BACKGROUND:**

Surgery, as the main approach in higher stages of gynecomastia, has different techniques regarding the staging of the disease. The more the grade of gynecomastia, the more complicated the used surgical techniques, conventionally. This study assessed the success rate of the simplest surgical technique in higher grades of gynecology as well as the satisfaction rate in patients and surgeon to offer using the technique for higher grades of the disease.

**METHODS:**

To evaluate the success and the satisfaction rates of periareolar incision and liposuction among patients with grade II and III gynecomastia, this cross-sectional study was conducted.

**RESULTS:**

The satisfaction rate was the main concern of the present study. The patients had a mean satisfaction score of 8.1±1.396 with the range of 5-10 from total 10 score. The majority of the patients expressed their satisfaction by 9 score. The total mean of physician satisfaction score was 8.36 at all levels.

**CONCLUSION:**

Like aesthetic reasons which lead individuals to seek solutions for their annoying gynecomastia, aesthetic satisfaction is a prominent concern for people who undergo surgical approach. So, the least surgical scar and complications are absolutely the most area of focus in this regard.

## INTRODUCTION

Gynecomastia is defined as a benign increased fibroglandular tissue in male breast more than 2 cm which is much palpable under the nipple and areola. This condition is more prevalent in Juvenile and elderly people.^1^ Global data showed a 32-36% prevalence worldwide.^2^ The mechanism refers to a benign cell infiltration and glandular cell proliferation as the main occurrence in male breast^3^^-^^5^ which is idiopathic in 25%.^6^ Medications like H_2_-blockers and anabolic drugs, particularly in athletes, are responsible for 10-20% of determined etiology before some medical conditions such as malnutrition and cirrhosis (8%), primary hypothyroid (1.5%) and renal diseases (1%).^3^

Generally, gynecomastia is somehow a consequence of an imbalance between androgens and estrogens in males.^7^^,^^8^ During first 1-2 years, the disease is too slow to develop and could be retreated using drugs before collagen fibers start to agglomerate around glandular tissue and ducts to run a fibrosis and hyalinization in breasts in more developed cases.^9^^-^^11^ This is the stage just treatable by surgery, as authors indicate. Surgery, as the main approach in higher stages of gynecomastia, has different techniques regarding the staging of the disease. The more the grade of gynecomastia, the more complicated the used surgical techniques, conventionally. 

Otherwise, surgery has its own complications and limitations. For instance, pedicle nipple reconstruction as well as free nipple technique always has the concern of the excessive remaining skin on the chest due to breast tissue excision which, in turn, forces surgeons to use extensive and long incisions and remove the skin at the area of surgery leaving large scars on chest. Other complications lie also beneath; such as nipple necrosis, areolar necrosis, hypoesthesia and other chronic problems. This is why we try the simplest technique in grade II and III of the disease, periareolar incision along with liposuction, which is used often for grade I gynecomastia.

This study assessed the success rate of the simplest surgical technique in this regard as well as the satisfaction rate in patients and surgeon to offer using the technique for higher grades of the disease. Decreased tissue trauma, faster and simpler surgery, and shorter hospital stay are the most popular advantages. Liposuction helps the chest wall symmetry and smoothness via skin which facilitates skin retraction with no obvious scar after a while.

## MATERIALS AND METHODS

Referring to the charts of the cases who underwent periareolar surgery plus liposuction , all the records between 2011 and 2013 were enrolled regarding inclusion and exclusion criteria. All the procedures were done in Imam Khomeini Hospital in Tehran, Iran, as the referral center for plastic surgery. The charts which contained no informed consent signed by patients and the patients who did not refer to be followed up in 6 months from surgery were excluded.

A questionnaire was provided to include chart records such as demographics; general, family, drug and medical history; reasons to surgery; outcomes; complications; and other surgery information. Complications and problems faced during surgery like bleeding, anatomical disorders, anesthetic insufficiency, and unusual drug use were also recorded. Acute consequences like pain, fever, bleeding, etc. were checked after surgery. Visual analogue scale (VAS) was widely used for pain rating with scores 0-10 which mean “no pain” and “extraordinary pain”, respectively.

Numbness, hypoesthesia and any sensory changes, breast size change, motion limitation, limited physical activity , surgical scar size, complete or partial areolar necrosis, chest wall asymmetry, auxiliary fat condition, and excess and sagging skin as well as patient’s shame to be in popular places were also checked in the studied charts as the prominent consequences of surgery. Satisfaction was rated using a similar system for pain scoring which contained score “0” to express no satisfaction and “10” to say that patient and/or surgeon got the best result of the surgery.

Gynecomastia was graded scaling system which contained three to four known grades. Grade I gynecomastia was the condition of accumulation little fat around the areolar area of breast which was palpable but did not shift the areola out of its anatomical position on the cross point of the major pectoralis muscle and the fifth rib on the chest wall. In grade II, the areola and inframammary fold (IMF) were below their anatomical normal horizontal level due to much fat and fibroglandular tissue. Pedicle nipple reconstruction surgery was usually advised to approach to this grade as a choice.

In terms of the most severe condition, much more fat and fibroglandular tissue were present under the breast and above person’s abdomen in addition to more dislocation in areola and IMF position in grade III of gynecomastia. This grade is usually corrected by free nipple surgical technique. In this study, the simplest technique was assessed for subjective and objective satisfaction referring patients and surgeons to use it instead of more complex techniques which was used ordinarily for grades II and III of gynecomastia. 

First, patients weere asked to stand upright to assess the excessive tissue and determine the area of surgery by markers, especially the incision line at sub-areolar border between hours 3 and 9. Our patients used general anesthesia after getting supine position in operating room with bilateral extended upper extremities before disinfection process in chest and upper abdomen. Diluted 50 ml of 1% lidocaine in 1 lit of ringer lactate solution was infused intravenously after antibiotic prophylaxis before starting the marked line to incise. Fibroglandular tissue was removed leaving 2-3 cm of the tissue beneath the nipple and areola and 1 cm tissue thickness under the breast skin. 

Tissue removal was done by a blade which separated the fat from the skin in anterior and from the major pectoralis muscle in posterior face. Then, liposuction was started from deepest layers towards superficial ones between sub-clavicular area and the 10^th^ rib through the incision described before. Liposuction used a 4 mm-diameter cannula while its sharp edge was held inward with no connection to muscles. This was to protect the skin and subcutaneous compartments from suction. Pinch test was used to check chest wall symmetry after enough tissue and fat removal by the surgeon before providing an elastic bandage fixed for 24 hours after surgery. This elastic bandage was usually changed into standard compressing vest after a day. Patients were always advised wearing the vest for 6 weeks, continuously and take it off only for taking shower.

Chi-square test and independent t-test were used to report frequencies by SPSS for windows (Version 21, Chicago, IL, USA). We considered a 95% confidence interval and significance with P value < 0.05. All the patients’ charts were checked, at first, for informed consents to allow the data to be used by researches as the first inclusion criterion. Patients who agreed to be visited by the researchers were examined and asked for acute or chronic surgery complications and their satisfaction. All the private information were safely kept by the principal investigators as patients’ secrets.

## RESULTS

**Fig. 1:**
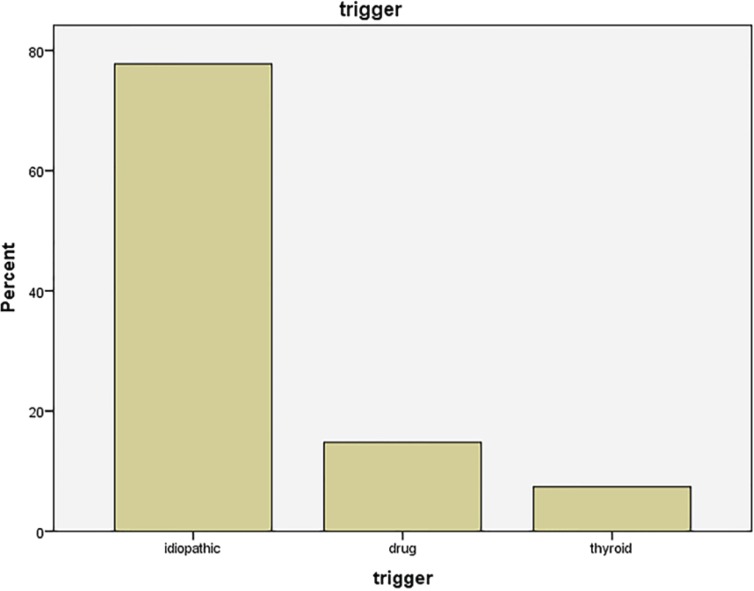
The etiologies and their frequencies for gynecomastia among our patients

During 2011-2013, twenty seven candidates were enrolled in the study. The mean age was 24.52±5 years with the mode of 18 years. The patients’ age ranged 17-36 years and the majority suffered from gynecomastia for 24 months. The mean time of disease was 40±23.8 months. (12-96 months). Out of 27, 14 had gynecomastia in grade II while 13 (48.1 %) were in grade III. For probable etiology of gynecomastia, 21 (77.87) got idiopathic origins of the disease while drugs were blamed for 4 individuals, while 2 patients had a history of hypothyroidism. Family history was positive in 8 patients (29.6%). Table 1 and 2 and figure 1 summarizes the data.

**Table 1:** Demographics, disease and procedure information

		**Age**	**Disease duration**	**Pain score**	**Surg duration**	**Patient** **satisf**	**MD1** **satisf**	**MD2** **satisf**	**MD3** **satisf**
N	Valid	27	27	27	27	27	27	27	27
	Missing	0	0	0	0	0	0	0	0
Mean		24.5185	40.0000	4.5926	134.8148	8.1111	8.5926	7.9259	8.5556
Mode		18.00^a^	24.00	3.00^a^	120.00	9.00	8.00	8.00^a^	9.00
Std. Deviation		4.94096	23.76811	1.90665	29.62852	1.39596	.74726	.95780	.97402
Minimum		17.00	12.00	1.00	90.00	5.00	7.00	6.00	6.00
Maximum		36.00	96.00	8.00	210.00	10.00	10.00	9.00	10.00

MD1: The Surgeon; MD2: The principal investigator; MD3: The fellow of plastic surgery.

**Table 2:** The frequency of the grades and etiologies of gynecomastia

**Etiology**	**Frequency (%)**	**Grade**	**Frequency (%)**
Idiopathic	21 (77.8)	II	14 (51.9)
Medication	4 (14.8)	III	13 (48.1)
Hypothyroid	2 (7.4)		

**Fig. 2:**
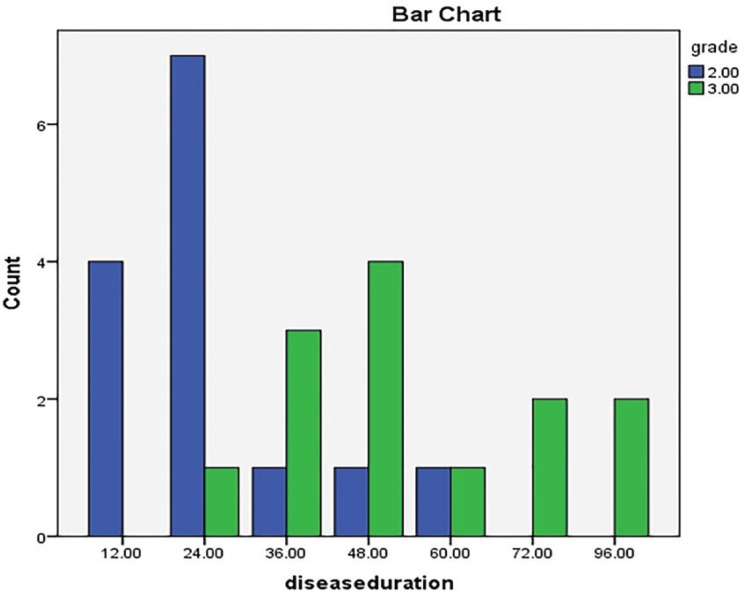
Correlation between the grade and chronicity of gynecomastia. (2=gradeII; 3=gradeIII

Since disease chronicity can develop the disease grade, we tried to evaluate any correlation between the time of involvement from and the grade of gynecomastia and interestingly found a positive relationship in this regard (*p*=0.018). Figure 2 shows the frequency of the grades regarding the lasting time of the disease. The mean surgery time was 82.8±17.6 minute which ranged 70-100 minutes. Twenty one patients stayed at hospital for 2 days after surgery but the rest were discharged 3 days after their surgery. So the mean postoperative hospital stay was 2.22±0.42 days with no correlation with operation time (*p*=0.85). The operation time was neither correlated to the grade of the disease (*p*=0.30) even by Fisher’s exact test.

**Fig. 3:**
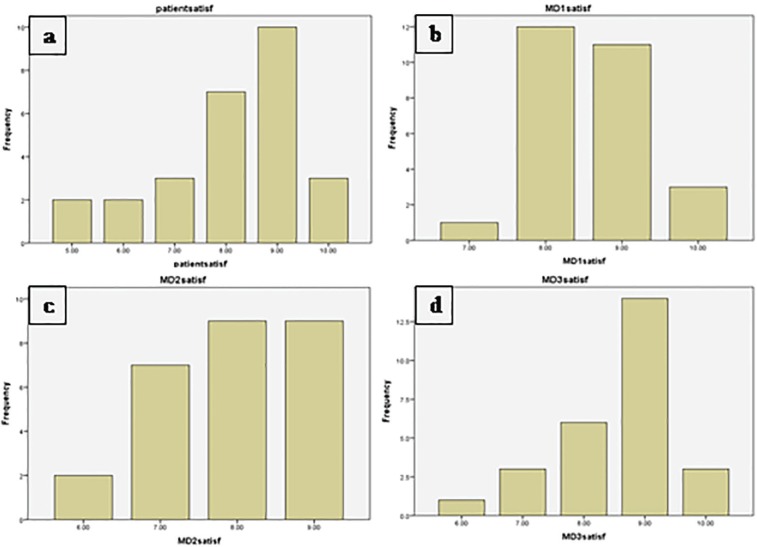
Satisfaction scores reported by the patients (a) and three physicians (b-d

The satisfaction rate was the main concern of the present study. The patients had a mean satisfaction score of 8.1±1.396 with the range of 5-10 from total 10 score. The majority of the patients expressed their satisfaction by 9 score. Physicians gave also their satisfaction score at three levels including the surgeon, the principal investigator of the present research, and the fellow of plastic surgery who resided in the ward. The surgeon’s score was 8.59±0.75 (7-10) in average with the mode of 8 of 10. The researcher faculty member gave 9/10 to the surgeries. The total mean of physician satisfaction score was 8.36 at all levels as can be seen in table 1 and figure 3. There was no linear correlation between the grade of the disease and satisfaction score of the surgery at all (*p*=0.17).

When surgical complications were concerned, 7 (25.97) and 2 patients (7.4%) complained from sensory changes and hypoesthesia in areola, respectively. The breast size, after surgery, satisfied 25 patients to get 92.6% total success rate. All patients, but 2 (7.4%) expressed their happiness of experiencing no excessive chest wall skin 6 months later when visited. Three patients confessed that they were not 100% satisfied by their appearance, finally (11.12%) which was totally different from their satisfaction from their breast size. Unpleasant surgical scars occurred in one (3.7%) subject, at the physicians’ point of view while partial areolar necrosis was noticed in 4 (14.8%) individuals. Chest wall asymmetry was seen in only one (3.7%) case. Auxiliary fat was successfully removed in 26 (96.3%) patients. 

## DISCUSSION

This study headed to assess the efficacy and outcome of periareolar incision technique in grades II and III of gynecomastia, which are usually corrected by other techniques with wider area of procedure. The minimum invasion of this technique would make it more acceptable if has even the same outcomes and complications of the advanced ones in higher grades of gynecomastia. Twenty seven individuals and total 53 breasts underwent surgery through the studied time period by the current performance.

In a similar study in the Northern Staffordshire Hospital during 2001-2009, 29 male patients were studied.^12^ The researchers evaluated the outcome of three procedures including only liposuction, only excisional surgery, and the combination of them to find 37.9% of patients who called their surgery perfect, while our patients presented 92.6% success rate and 89% accuracy. The satisfaction rate was 96.5% totally containing perfect, good and satisfying cases, which is not much more than ours (92.6%). Furthermore, 100% of our patients were completely satisfied by the absence of excessive skin on their chest wall after 6 months of surgery in addition to 96% success rate in auxiliary fat removal. 

Through a different incisions (criss-cross incision right on the nipple), a similar technique was introduced to remove fibroglandular tissue along with liposuction like our report.^13^ Between 2012 and 2013, 28 cases were treated through two 6-7 mm incisions on both sides of the chest using number 4 liposuction canullae. They started the procedure with liposuction before making the criss-cross incision and this is unlike our experience which conducted liposuction through the only periareolar incision. So we could get rid of two scars on both sides of patient’s chest. Results showed that all 28 their patients were satisfied by the outcomes.

In terms of postoperative excessive skin on the chest wall and its aesthetic points, children were shown to have more skin retract ability in comparison to older people. This fact lead the researchers to conduct a retrospective study on patients who were younger than 18 years and got surgical approach for their idiopathic gynecomastia. The majority of their patients had the grade III of the disease and the lower rate was reported for grade II (46% and 40.5%). Subcutaneous mastectomy and liposuction were done in 26 out of 37 patients (70%). They concluded better results for the combination of mastectomy and liposuction with more aesthetic satisfaction, probably because of selecting children.

In other study, Wolter *et al.*^15^ used water-jet assisted liposuction along with periareolar mastectomy in patients who had grades I-III by Simon’s classification. For II_b_, they used circumferential mastopexy in addition to the above technique and inferior pedicled mammoplasty was applied for grade III. The most satisfaction rate was reported 88% by their study which is too low comparing ours, although they used globally advised techniques for each grade of gynecomastia.

Kasielska *et al.* showed the best results of surgical approach for subcutaneous mastectomy using circumareolar incision without liposuction in terms of aesthetic affairs.^16^ Brafa *et al.* used periareolar inferior or inverted “omega” incision and circumareolar incision and liposuction in their experience for surgical approach to gynecomastia in 126 candidates between 2000 and 2006. Their average satisfaction score was 8.2/10 with a complication rate of 17.72% which is comparable to our rates.^17^

To conclude, like aesthetic reasons which lead individuals to seek solutions for their annoying gynecomastia, aesthetic satisfaction can be a prominent concern for people who undergo surgical approach. So, the least surgical scar and the least complications is absolutely the most area of focus in this regard. 

## CONFLICT OF INTEREST

The authors declare no conflict of interest.
